# Identification of long non-coding RNA signatures for squamous cell carcinomas and adenocarcinomas

**DOI:** 10.18632/aging.202278

**Published:** 2020-12-09

**Authors:** Suyan Tian, Mingbo Tang, Jialin Li, Chi Wang, Wei Liu

**Affiliations:** 1 Division of Clinical Research, First Hospital of Jilin University, Changchun 130021, Jilin, P.R. China; 2 Department of Thoracic Surgery, First Hospital of Jilin University, Changchun 130021, Jilin, China; 3 Department of Internal Medicine, College of Medicine, University of Kentucky, Lexington, KY 40536, USA; 4 Markey Cancer Center, University of Kentucky, Lexington, KY 40536, USA

**Keywords:** long non-coding RNA (lncRNA), gene signature, squamous cell carcinoma (SCC), adenocarcinoma (AC), pan-cancer analysis

## Abstract

Studies have demonstrated that both squamous cell carcinomas (SCCs) and adenocarcinomas (ACs) possess some common molecular characteristics. Evidence has accumulated to support the theory that long non-coding RNAs (lncRNAs) serve as novel biomarkers and therapeutic targets in complex diseases such as cancer.

In this study, we aimed to identify pan lncRNA signatures that are common to squamous cell carcinomas or adenocarcinomas with different tissues of origin. With the aid of elastic-net regularized regression models, a 35-lncRNA pan discriminative signature and an 11-lncRNA pan prognostic signature were identified for squamous cell carcinomas, whereas a 6-lncRNA pan discriminative signature and a 5-lncRNA pan prognostic signature were identified for adenocarcinomas. Among them, many well-known cancer relevant genes such as MALAT1 and PVT1 were included.

The identified pan lncRNA lists can help experimental biologists generate research hypotheses and adopt existing treatments for less prevalent cancers. Therefore, these signatures warrant further investigation.

## INTRODUCTION

Squamous cell carcinomas (SCCs) are neoplasms of the squamous cells that compose most of the skin’s upper layers (epidermis). They may also occur in other tissues, including mouth, esophagus, bladder, prostate, lung, vagina and cervix. Studies [1–3] have suggested that regardless of the tissue of origin, SCC patients share some common molecular characteristics, which thus, may be clustered together. The statistical analyses were carried out using either an integrative analysis including several omics data types or a single analysis on mRNA expression profiles alone. For example, using the cluster-of-cluster-assignments method [4], an integrated dataset including 6 types of omics data for 12 human solid cancer types was analyzed. Results showed that one of the clusters (the squamous-like category) was dominated by lung squamous cell carcinoma and head and neck squamous cell carcinoma [2], in spite of both originating in distinct organs. Naturally, it is anticipated that a pan-gene signature that is commonly applicable to all SCCs may exist.

On the other hand, adenocarcinoma (AC) is a type of cancer that starts in the mucous glands inside of organs, including lungs, colon, esophagus, prostate or even breasts. The Cancer Genomic Atlas (TCGA) network [3] has demonstrated that esophageal adenocarcinoma resembles gastric adenocarcinoma more than it resembles esophageal squamous cell carcinoma (ESCC).

Genome-wide transcriptome analysis has revealed that non-protein-coding genes, which once were regarded as evolutionary junk, account for about 98 % of the human transcripts. Long non-coding RNAs (lncRNAs) are a major class of non-coding RNAs that have a length of more than 200 nucleotides [5]. Nowadays, significant evidence has accumulated to support the theory that lncRNAs can serve as novel biomarkers and therapeutic targets in complex diseases such as cancer [6]. According to Ching et al. [7], lncRNAs are more tissue specific than mRNAs. Nevertheless, in the literature it is easy to find many lncRNAs that are associated with several cancer types that start at different sites/organs. For example, metastasis associated lung adenocarcinoma transcript 1 (MALAT1), a well-known oncogene, has been linked with a variety of cancers such as pancreatic cancer [8–10], prostate cancer [11, 12], hepatocellular carcinoma [13] and thyroid cancer [14]. Specifically for SCCs, MALAT1 has been verified experimentally to correlate with esophageal [15, 16] and oral SCC [17]. Similarly, HOTAIR has been validated by experiments to be correlated with lung adenocarcinoma [18, 19] and gastric carcinoma [20] in addition to other cancer types according to the lncRNAdisease2.0 knowledgebase [21]. Therefore, it is reasonable to speculate the existence of common lncRNAs that play essential roles in many cancer types including SCCs and ACs despite the fact that lncRNAs are more tissue specific than mRNAs. To the best of our knowledge, no study has yet explored the existence of lncRNA signatures for either a pan SCC type or a pan AC type.

Similar to other high-dimensional omics data, feature selection is usually exploited when constructing lncRNA signatures. The goals of feature selection are to eliminate the curse of dimensionality issue, speed up the learning process, avoid over-fitting and thus generate more reliable discriminative or prognostic gene lists/signatures. The selection of relevant lncRNA lists can be realized by using a penalized/regularized regression model, which belongs to the family of embedded feature selection methods. As opposed to filter methods, the embedded methods take the joint effects of covariates into account, and thus can model gene dependencies and concordance. On the other hand, such methods have better computational complexity than the wrappers methods. Therefore, a penalized regression model has harnessed increasing attention from many statisticians and computational biologists [22].

In this study, pan lncRNA signatures commonly applicable to SCCs or ACs with different tissues of origin were constructed with the aid of elastic-net regularized regression models. Specifically, an extensive investigation of potential discriminative and prognostic gene signatures was sought in TCGA, where both pieces of information are available. While a gene signature that can distinguish normal controls from tumors may provide insightful clues on initiation and development of the disease, a prognostic gene signature focuses more on the prediction of disease progression, thus facilitating more effective interventions for patients with poor prognosis to prolong their survival or cure the disease. Therefore, both types of signatures are of crucial importance.

## RESULTS

### Discriminative lncRNA signatures

### Squamous cell carcinomas

Separate logistic elastic-net regression models were fit for the LUSC and HNSC studies (the CESC study were excluded due to the non-availability of normal tissues). A 173-lncRNA discriminative signature for HNSC and a 277-lncRNA signature for the LUSC study were identified. The gene lists for these two studies resulted in 35 overlapped lncRNAs (Table 1). Of those, 10 genes have been reported in the literature to correlate to one specific cancer type or more using real experiments (rather than being predicted using a computational method) according to the lncRNADisease 2.0 database.

**Table 1:** Pan discriminative lncRNAs for the squamous cell carcinoma type.

**Symbols**	**Target mRNA**	**Cancer types (experimentally validated)**	**Recent publications recording associated cancer types**
WDFY3-AS2	WDFY3		Ovarian [23]; ESCC [24]; LUAD [25]; breast [26, 27]; HCC [28]
CFAP99			
DUXAP8		Stomach	HCC [29–31]; colon [32, 33]; bladder [34, 35]; pancreatic [36]; ESCC [37]; esophageal [38]; renal cell carcinoma [39, 40]; NSCLC [41]
FIRRE			Diffuse large B-cell lymphoma [42]; colon [43]
HAGLR	EVX2, HOXD13, HOXD12, HOXD11, HOXD10, HOXD9, HOXD8, HOXD3, AC009336.2, HOXD4, HOXD1, MTX2	Stomach, cervical, NSCLC, neuroblastoma, glioma, ovarian, urinary bladder, HCC, prostate, thyroid	Esophageal [44]; LUAD [45]; colon [46]
LOC101929331			
DUXAP10		Ovarian, colorectal	Myeloid leukemia [47]; ESCC [48]; bladder [49]
ABHD11-AS1	VPS37D, DNAJC30, BUD23, STX1A, ABHD11, CLDN3, CLDN4, METTL27	Urinary bladder, stomach	Thyroid [50, 51]; pancreatic [52, 53]; endometrial carcinoma [54]; ovarian [55]; colon [56, 57]
LOC101928118			
SLC16A1-AS1	SLC16A1, LRIG2	Cervical, lung, astrocytoma	NSCLC [58]; OSCC [59]; HCC [60]
MFI2-AS1	MFI2		Colon [61]; glioma [62]; HCC [63]
LINC00443	ARGLU1		Renal cell carcinoma [64]
DLEU2	SPRYD7, TRIM13, KCNRG	Lymphoma, laryngeal, leukemia, pancreatic, astrocytoma	Gastric [65]; NSCLC [66, 67]; pancreatic [68]; HCC [69]; esophageal [70]
LOC101927596			
LOC105375401			
LOC100128164			
ADAMTS9-AS1	ADAMTS9	Ovarian epithelial cancer, malignant glioma	Prostate [71]; COAD [72]
ADAMTS9-AS2	ADAMTS9	Malignant glioma, renal, NSCLC	Ovarian [73]; gastric [74–76]; glioblastoma [77]; breast [26, 78]; TSCC [79]; esophageal [80]
LINC00996	LRRC61, RARRES2, REPIN1, AC073111.3, ZNF775, AC073111.5, GIMAP8, GIMAP7		
LINC01296		Prostate, stomach, urinary bladder, colorectal	Osteosarcoma [81]; neuroblastoma [82]; ESCC [83, 84]; NSCLC [85]; HCC [86]; breast [87]; ovarian ([88])
LOC101929066			
LOC101929595			
LOC440028			
LOC101929340			
LINC00958			Cervical [89, 90]; OSCC [91, 92]; NSCLC [93]; pancreatic [94]; nasopharyngeal [95]; HNSCC [96]; HCC [97]; gastric [98]
LOC101927392			
RBPMS-AS1	RBPMS		
BBOX1-AS1	FIBIN, BBOX1		Colon [99]; cervical [100]
TMPO-AS1	TMPO, SLC25A3, IKBIP	Astrocytoma	
GS1-120K12.4			
TTC39A-AS1	RNF11, TTC39A, EPS15		
LOC400568			
TMEM220-AS1	MYH3, SCO1, ADPRM, TMEM220, TMEM238L, PIRT		
LDLRAD4-AS1			
LINC00551	EFNB2, ARGLU1		

* Cancer types (with experimental validations) to be associated with that specific lncRNA according to the lncRNAdisease2.0 database.

** Only a few examples are given since the number of relevant publications is large.

COAD: colon adenocarcinoma; ESCC: esophageal squamous cell carcinoma; HCC: Hepatocellular carcinoma; HNSCC: head and neck squamous cell carcinoma; LUAD: lung adenocarcinoma; NSCLC: non-small cell lung cancer; OSCC: oral squamous cell carcinoma; TSCC: tongue squamous cell carcinoma.

### Adenocarcinomas

Among the AC cohort, separate logistic elastic-net regression models identified a 185-lncRNA signature for the LUAD study, a 173-lncRNA signature for the STAD study and a 114-lncRNA signature for the PRAD study. The intersection of these three gene lists has 6 lncRNAs: UBXN10-AS1, SNHG20, ADAMTS9-AS1, ADAMTS9-AS2, PVT1 and VPS9D1-AS1 (Table 2). Of note, both ADAMTS9-AS1 and ADAMTS9-AS2 also belong to the 35-overlapped lncRNA list for SCC discriminative analysis.

**Table 2:** Pan discriminative lncRNAs for the adenocarcinoma type.

**Symbols**	**Target mRNAs**	Cancer types (experimentally validated)*	**Recent publications recording associated cancer types**
UBXN10-AS1	PLA2G5, PLA2G2D, PLA2G2F, PLA2G2C, UBXN10		
SNHG20	SEC14L1	HCC, ovarian, colorectal, NSLCL, stomach	Breast [101]; cervical [102]; bladder [103]; prostate [104]; ESCC [105]; OSCC [106, 107]; nasopharyngeal [108]
ADAMTS9-AS1	ADAMTS9	Ovarian epithelial cancer, glioma	Prostate [32]; COAD [72]
ADAMTS9-AS2	ADAMTS9	Glioma, renal, NSCLC	Ovarian [73]; gastric [74–76]; clear cell renal cell carcinoma [109]; glioblastoma [77]; breast [26, 78]; TSCC [79]; esophageal [80]; NSCLC [110]
PVT1	MYC	Colorectal, HCC, prostate, cervical, stomach, lung, esophageal and others	EAC [111] **
VPS9D1-AS1	DPEP1, CHMP1A, SPATA33, CDK10, SPATA2L, VPS9D1, ZNF276, FANCA	Stomach	NSCLC [112, 113]; prostate [114, 115]

*Cancer types (with experimental validations) to be associated with that specific lncRNA according to the lncRNAdisease2.0 database.

**Only one example is given since the number of relevant publications is large.

COAD: colon adenocarcinoma; EAC: esophageal adenocarcinoma; ESCC: esophageal squamous cell carcinoma; HCC: Hepatocellular carcinoma; NSCLC: non-small cell lung cancer; OSCC: oral squamous cell carcinoma; TSCC: tongue squamous cell carcinoma.

Furthermore, the number of overlaps between LUAD and STAD is 34; 18 between LUAD and PRAD and 11 between STAD and PRAD. All these overlaps took substantial proportions of the identified lncRNA signatures.

### Prognostic lncRNA signatures

### Squamous cell carcinoma

Cox elastic-net regression models selected a 462-lncRNA list for the LUSC study, a 597-lncRNA list for the HNSC study and a 263-lncRNA list for the CESC study, respectively. Among the 3 lists were 11 overlaps (Table 3), and 5 of them (CFLAR-AS1, SLC16A1-AS1, SIRPG-AS1, LOC389641 and LINC00593) were experimentally validated as cancer related genes according to the lncRNADisease 2.0 database. In Table 3, the target mRNAs of these 11 lncRNAs are given.

**Table 3:** Pan prognostic lncRNAs for the squamous cell carcinoma type.

**Symbols**	**Target mRNAs**	Cancer types (experimentally validated)*	**Recent publications recording associated cancer types with experimental validations**
SLC16A1-AS1	SLC16A1, LRIG2	Cervical, lung, astrocytoma	NSCLC [58]; OSCC [59]; HCC [60]
CFLAR-AS1	FAM126B, NDUFB3 CFLAR, CASP10, CASP8	ESCC, astrocytoma	
SPATA13-AS1	AL359736.1, SPATA13, C1QTNF9		
LINC00311	GSE1		Thyroid [117]
LINC01305	OLA1, SP9, CIR1, SCRN3		Cervical [118]; NSCLC [119]
LINC01399	ISX, HMGXB4, TOM1		
FGF14-AS1	FGF14		
SIRPG-AS1	SIRPD, AL049634.2, SIRPB1, SIRPG	Astrocytoma	
LOC389641	TNFRSF10D, TNFRSF10A, CHMP7, R3HCC1, LOXL2	PADA	PDAC [120]
LINC00593		Astrocytoma	
MKNK1-AS1	DMBX1, AL136373.1, KNCN, MKNK1, MOB3C, ATPAF1, TEX38		

*Cancer types (with experimental validations) to be associated with that specific lncRNA according to the lncRNAdisease2.0 database.

ESCC: esophageal squamous cell carcinoma; HCC: Hepatocellular carcinoma; NSCLC: non-small cell lung cancer; OSCC: oral squamous cell carcinoma; PDAC: pancreatic ductal adenocarcinoma.

### Adenocarcinoma

Cox elastic-net regression models identified a 53-lncRNA set for the STAD study and a 95-lncRNA list for the LUAD study. The PRAD study was excluded from the prognosis analysis since the number of events (deaths) was too small to guarantee a valid analysis. The intersection set of the two gene lists includes 5 lncRNAs (EIF1AX-AS1, LINC00115, LINC01237, MALAT1 and LINC00528), among which only LINC00115 and MALAT1 have been experimentally validated to correlate with cancers according to the lncRNADisease2.0 database. The associated cancer types of and the target mRNAs by these five lncRNAs given by the lncRNADisease 2.0 database are listed in Table 4.

**Table 4:** Pan prognostic lncRNAs for the adenocarcinoma type.

**Symbols**	**Target mRNAs**	Cancer types (experimentally validated)*	**Recent publications recording associated cancer types with experimental validations**
EIF1AX-AS1	MAP7D2, EIF1AX, RPS6KA3		
LINC00115	SAMD11	Astrocytoma, lung adenocarcinoma	Glioma [126]; breast [127]
LINC01237	GAL3ST2, NEU4, PDCD1, RTP5, AC131097.2		
LINC00528	BCL2L13, BID, MICAL3		LSCC [128]
MALAT1	FRMD8, SCYL1, LTBP3, SSSCA1, FAM89B, EHBP1L1, KCNK7, MAP3K11	Pancreatic, prostate, breast, gallbladder, OSCC, stomach, NSCLC, ESCC, and others	Breast [129]; ovarian [130]; glioblastoma [131] **

*Cancer types (with experimental validations) to be associated with that specific lncRNA according to the lncRNAdisease2.0 database.

**Only a few examples are given since the number of relevant publications is large.

ESCC: esophageal squamous cell carcinoma; LSCC: laryngeal squamous cell carcinoma; LUAD: lung adenocarcinoma; OSCC: oral squamous cell carcinoma.

### Performance evaluation

**Figure 1:**
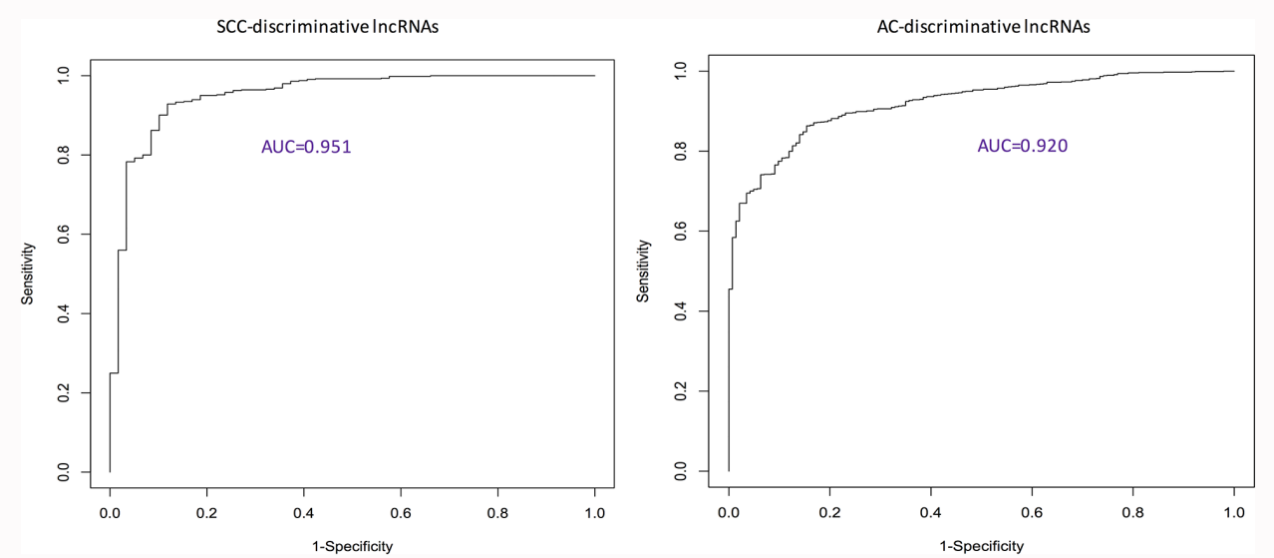
**ROC curves showing the performance of identified discriminative lncRNA lists.** AC: adenocarcinoma; AUC: area under the ROC curve; ROC: receiver characteristic operator; SCC: squamous cell carcinoma.

**Figure 2:**
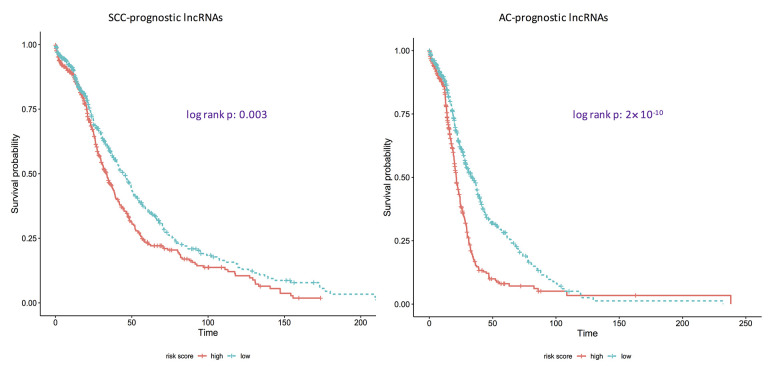
**Kaplan-Meier curves showing if identified prognostic lncRNA lists are associated with survival rates for adenocarcinomas and squamous cell carcinomas.** AC: adenocarcinoma; SCC: squamous cell carcinoma. p: the corresponding p-values of log-rank tests to test if the survival curves of high-risk group and low-risk group are same. Here, the ridge Cox regression models were used to estimate the coefficients of lncRNAs and then risk scores were calculated. Then the median value of risk scores was used as the cutoff to divide the AC/SCC patients into the high-risk group and the low-risk group.

Using ROC curves (Figure 1), the discriminative ability of identified pan-SCC and pan-AC lncRNA signatures was evaluated. The AUCs of these two signatures are 0.951 and 0.920 for the SCC type and the AC type, respectively. Overall, these two signatures perform well. According to the log-rank tests (Figure 2), the identified pan-SCC and pan-AC prognostic lncRNA signatures have good prognostic values as well.

### Enriched pathways

**Figure 3:**
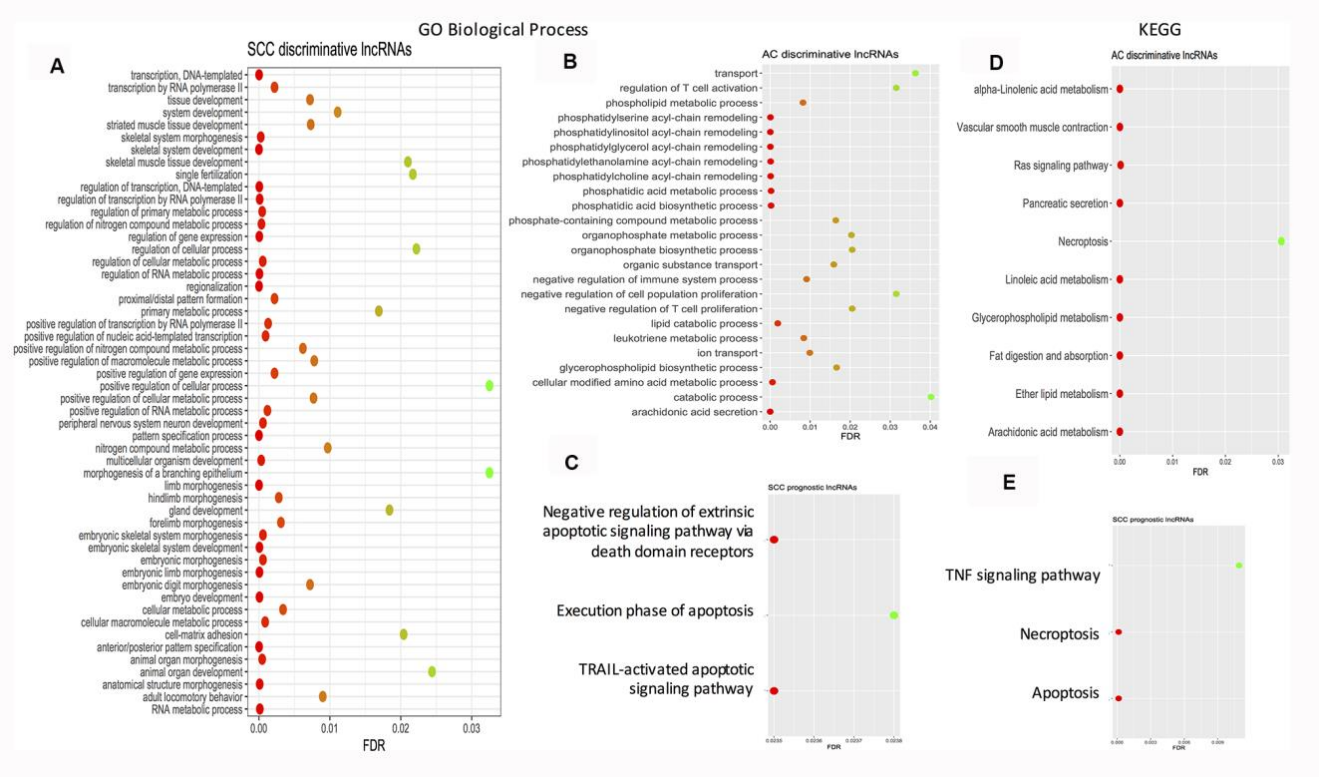
**Dot plots showing the enriched gene ontology biological process terms and KEGG pathways.** (**A**) Enriched GO biological process terms by SCC discriminative lncRNA signature. (**B**) Enriched GO biological process terms by AC discriminative lncRNA signature. (**C**) Enriched GO biological process terms by SCC prognostic lncRNA signature. (**D**) Enriched KEGG pathways by AC discriminative lncRNA signature. (**E**) Enriched KEGG pathways by SCC prognostic lncRNA signature. AC: adenocarcinoma; FDR: false discovery rate; GO: gene ontology; KEGG: Kyoto Encyclopedia of Genes and Genomes; FDR: false discovery rate; SCC: squamous cell carcinoma.

Using String software, pathway enrichment analysis was conducted. The results showed that no pathway or GO biological process term was enriched by the AC prognostic signature. Enriched pathways/GO biological process terms for the SCC and AC discriminative signatures and the SCC prognostic signatures are given in Figure 3. As far as the enriched KEGG pathways are considered, there is one overlap – necroptosis between the AC discriminative category and the SCC prognostic category. Necroptosis is a programmed caspase-independent cell death. Studies have shown that tumor undertakes necroptosis *in vivo* and the process has pro- or anti- tumor effects in cancer development and progression [116]. At the level of pathways, both SCCs and ACs may share some common features.

The overlapped proportions/ratios of these prognostic lncRNAs are substantially less than those taken by the discriminative lncRNAs, which may be explained by two reasons. First, many studies in the literature have pointed out that prognosis is more difficult than discrimination/diagnosis. For instance, for the LUAD patients at the same stage, distinct molecular subtypes with different prognoses exist. Correspondingly, prognostic gene signatures are anticipated to be more type-specific, while the sizes of these signatures are to be much larger. Indeed, several studies suggested that possibly dozens of genes can make a perfect segmentation of tumors and controls or of different subtypes, but this is not so for the segmentation of patients with good prognosis versus poor prognosis. Second, the overall survival time may not be a good surrogate for prognosis. Also, these AC and SCC cohorts may not be followed up for a period long enough to develop adequate events/deaths for an accurate survival analysis. With these two disadvantages, many false positives may be produced and included in the resulting gene lists.

## DISCUSSION

### Discriminative lncRNAs with high biological relevance to cancer

### Overlapped lncRNAs by both types

First, the focus is on the two overlapped lncRNAs – ADAMTS9-AS2 and ADAMTS9-AS1. According to the lncRNADisease 2.0 database [21], both lncRNAs are associated with several cancer types. In addition, a search of the PubMed database on recent investigation of the association between these two genes and cancer reveals that more studies concerned ADAMTS9-AS2. For example, a very recent study reported that the expression level of ADAMTS9-AS2 is lower in esophageal cancer tissues and over-expressing it can suppress the development of esophageal cancer via inducing CDHS promoter methylation [80]. The recently experimentally validated cancer types for this lncRNA included breast cancer [26] (using qRT-PCR), gastric cancer (by constructing a potential ceRNA network using bioinformatics tools and then validating the ADAMTS9-AS2/miR-372/CADM2 axis using qRT-PCR and dual luciferase reporter assay) [75], tongue squamous cell carcinoma (constructing ADAMTS9-AS2/miR-600/EZH2 ceRNA network) [79] and others. In contrast, only two recent studies on ADAMTS9-AS1 [71, 72] with experimental validations have been found.

### Pan-AC lncRNAs

Of the pan-AC specific lncRNAs, all except UBXN10-AS1 and VPS9D1-AS1 have been experimentally validated to be associated with a variety of cancer types according to the lncRNADisease 2.0 knowledgebase [21]. For example, both SNHG20 and PVT1 are related to stomach cancer. PVT1 is a well-known oncogene and correlated with a variety of cancers according to both the lncRNADisease 2.0 knowledgebase and the GeneCards database [121]. In our previous study [122], PVT1 was identified as a subtype-specific prognostic gene for esophageal adenocarcinoma using a feature selection algorithm called the Cox-filter method [123]. In this study, it was identified as a pan AC discriminative gene, which leads us to anticipate that PVT1 may be more relevant to the AC type than the SCC type, even though PVT1 has also been verified in the literature to associate with the SCC cancer type such as esophageal squamous cell carcinoma [124]. Another feasible explanation is that PVT1 may be a pan-gene commonly applicable for both SCCs and ACs. Further investigation is warranted. Also, SNHG20 is regarded as vital in many cancers [125] and identified as a subtype-specific prognostic gene for laryngeal squamous cell carcinoma in our previous study [36] using a computational method. Recent studies have experimentally validated that this gene is related to esophageal squamous cell carcinoma [105], nasopharyngeal carcinoma [108] and oral squamous cell carcinoma [106–107]. Again, the existence of pan-genes for both AC and SCC types may explain this to some extent.

In addition to gastric cancer as indicated by the lncRNADisease 2.0 database, non-small cell lung cancer [112–115] and prostate cancer have been linked to VPS9D1-AS1 by several recent experimental studies.

### Pan-SCC lncRNAs

DUXAP8 was recently identified as a pan-cancer gene using meta-analysis and TCGA pan-cancer data [30]. Focusing on hepatocellular carcinoma, the authors did a qRT-PCR experiment to verify the diagnostic and prognostic values of this gene for cancer patients. The results showed that the expression value of DUXAP8 increased in tumor tissues when compared with their paired normal tissues. In the meantime, high expression of this gene is related to a poor prognosis. In addition to this study, this lncRNA has been linked to several other cancer types, renal cell carcinoma [40] and colon cancer [33], for example. For SCCs specifically, it is linked to esophageal squamous cell carcinoma [37].

Additionally, HAGLR was listed as being highly relevant to cancer. Specific for the SCC or AC cancer type, based on the data analysis of MTT assay, qRT-PCR and western blot experiments, Lu et al. [132] showed that the expression levels of HAGLR were associated with non-small cell lung cancer tumor lymph node metastasis status, stage, and overall survival. With inhibition of HAGLR in non-small cell lung cancer cells, cell proliferation and invasion can be suppressed. Also, Yang et al. [133] showed that down-expression of HAGLR inhibited LAMP3 expression by sponging miR-143-5p and suppressed the progression of esophageal carcinoma.

### Prognostic lncRNAs with high biological relevance to cancer

### Pan-SCC lncRNAs

Interestingly, SLC16A1-AS1 is deemed as both a prognostic gene and a discriminative gene for the pan-SCC type. Some markers may play crucial roles in both diagnosis and prognosis of a disease, and in our opinion such markers deserve more attention since targeting them may not only prevent occurrence of the disease but also reverse the consequences of the disease after disease onset.

A search of the PubMed database revealed several articles that describe the association between this lncRNA and cancer. For example, Liu et al. [58] demonstrated that SLC16A1-AS1 was under-expressed in non-small cell lung cancer tissues and cell lines with an *in situ* hybridization experiment. They also showed that SLC16A1-AS1 overexpression could functionally inhibit the viability and proliferation of lung cancer cells, block the cell cycle and promote cell apoptosis *in vitro*.

It is worth pointing out that none of the 11 pan-SCC prognostic lncRNAs are under comprehensive investigation according to lncRNADisease 2.0. In the PubMed search for recent publications exploring the association of these lncRNAs with cancer, only several additional links were harnessed. For instance, one study indicated that LINC01305 was related to cervical cancer [118] and the other [119] associated it with non-small cell lung cancer.

### Pan-AC lncRNAs

Of the pan-AC prognostic lncRNAs, MALAT1 is a well-known oncogene and has been linked to a variety of cancers so far, including non-small cell lung cancer, cervical cancer, tongue squamous cell carcinomas and gastric cancer. In our previous study [122], it was identified as a subtype-specific prognostic gene for laryngeal squamous cell carcinoma using the Cox-filter method [123], further implying its possibility of being a pan-cancer lncRNA.

As far as LINC00115 is concerned, the lncRNADisease 2.0 database linked it to astrocytoma and lung adenocarcinoma. Two very recent studies [126, 127] have added breast cancer and glioma to this list. On the other hand, LINC00528 has recently been demonstrated to relate to laryngeal squamous cell carcinoma [128]. The respective recent publications exploring the association of these lncRNAs with cancer in the PubMed database are summarized in Tables 1–4.

Given the promising results that a substantial proportion of pan-SCC/AC genes identified by the bioinformatics procedure are related to a variety of cancer types and thus have a good biological relevance, the overlapped discriminative- and prognostic- lncRNAs warrant further investigation.

## CONCLUSIONS

In this study, discriminative and prognostic lncRNA lists for pan SCC and pan AC types were constructed using first elastic-net regression models to obtain individual lncRNA lists for each cancer study, and subsequently identifying the intersection of the resulting lists.

Given the fact that the research on lncRNAs has been a hot topic in the past several years, the shortage of lncRNA markers for complex diseases such as cancer, especially prognostic ones (since the outcome is survival time which needs a long period of follow-up to collect) is still common. The identified lncRNA lists in this article may help experimental biologists generate research hypotheses and design their own experiments correspondingly.

It is worth pointing out that pan cancer gene signatures are equal in importance to type-specific gene signatures. While with the type-specific signature a better prediction for progression and prognosis is possible, some existing drugs/therapies for other cancers may be adopted to treat a less prevalent cancer. Such adoption may help save resources and time for developing a brand-new drug for one specific cancer type, and therefore may increase the chance of survival for cancer patients.

## MATERIALS AND METHODS

### Experimental data

The Atlas of ncRNA in Cancer (Tanric) database [134] included lncRNA expression profiles (RNA-Seq data) for 20 cancer types in the TCGA project. We identified 8 cancers that are either the SCC type or AC type with 100 % confidence: cervical squamous cell carcinoma (CESC), head and neck squamous cell carcinoma (HNSC) and lung squamous cell carcinoma (LUSC), lung adenocarcinoma (LUAD), prostate adenocarcinoma (PRAD), stomach adenocarcinoma (STAD), colon adenocarcinoma (COAD), and rectum adenocarcinoma (READ). For the colon and rectum cohorts, no normal tissues were provided and the number of deaths was also very small, rendering both the discrimination and prognosis analyses less trustworthy or even impossible. Consequently, these two cohorts were excluded, and the final SCC study included cervical, head and neck, and lung SCC cohorts. The AC cohorts included lung, prostate and stomach.

For the 6 cohorts, expression profiles were downloaded fromtheTanricwebpage(https://ibl.mdanderson.org/tanric/_design/basic/download.html). Corresponding clinical information such as overall survival time, American Joint Committee on Cancer staging status and age were downloaded from TCGA’s Genomic Data Commons (http://www.cbioportal.org/). Tanric [134] includes expression profiles of 12,727 lncRNAs quantified as the RPKM (fragments per kilo-bases per million) counts. Table 5 summarizes clinical characteristics of these six studies.

**Table 5:** Clinical characteristics of squamous cell carcinoma studies and adenocarcinoma studies.

	**# of patients(stages I-IV)**	**# of deaths**	**Missing of survival time/Missing of stage information/ No. of all zero expression**	**# of normal tissues**
Squamous Cell Carcinoma				
Head and Neck	426 (24/63/73/200)	187	1/66/43	42
Lung	220 (116/55/43/4)	105	4/2/17	17
Cervical^1^	196 (--/--/--/--)	42	0/--/220	3
Adenocarcinoma				
Lung	488 (262/112/81/23)	174	9/10/76	58
Stomach	285 (39/99/103/23)	94	3/21/10	33
Prostate^2^	374 (--/--/--/--)	6	0/--/53	52

--: not available.

^1^Since no stage information is available and there are only 3 normal tissues, this cohort was excluded from and discriminative analysis.

^2^The prostate cohort was not included in the cancer-type specific analysis for survival analysis since the number of events/deaths is small. In summary, 2 studies were used for SCC discriminative analysis and 3 for survival analysis, while for AC 3 studies were used for discriminative analysis and 2 for survival analysis.

Next, ensemble IDs were mapped into gene symbols and lncRNAs without a valid gene symbol were deleted, leaving 3,173 lncRNAs retained for further analysis. Lastly, the RPKM were logarithm transformed (base 2) after being added 1s (in order to avoid having log transformation on zeros), to make the distribution of resulting lncRNA expression values approximate to a normal one.

### Statistical methods

### Elastic-net regularized regression

A logistic elastic-net regularized regression model and a Cox elastic-net regularized regression model were fit for each cohort to identify the respective discriminative lncRNA signature and prognostic lncRNA signature for the specific cancer type. Briefly, elastic-net penalty is a linear combination of L1 (LASSO) and L2 (ridge) penalties. It is well known that the L1 penalty introduces sparseness into the model by offsetting the coefficients of insignificant genes to zeros. In contrast, the L2 penalty introduces a grouping effect by smoothing the coefficients of correlated genes to a common value. In this sense, it can also penalize large β coefficients. Therefore, the elastic-net penalty incorporates the advantages of both L1 and L2 penalties, i.e., being capable of feature selection and giving the grouping structure some consideration. The mathematical notation is,

$$\lambda (\alpha |\beta {|}_{1}+(1-a)||\beta |{|}_{2})$$

Here, the parameter controls the ratio of L1 penalty to L2 penalty. The tuning parameter λ determines the amount of regularization used, with a large value corresponding to a heavy penalization on β coefficients and a small value to a light one. Its optimal values were determined using 10-fold cross-validations.

Depending on the type of outcome, it may be combined with the corresponding objective function to frame into a variety of regularized regression models such as a logistic elastic-net regression for a binary outcome and a Cox elastic-net regression for survival time.

**Figure 4:**
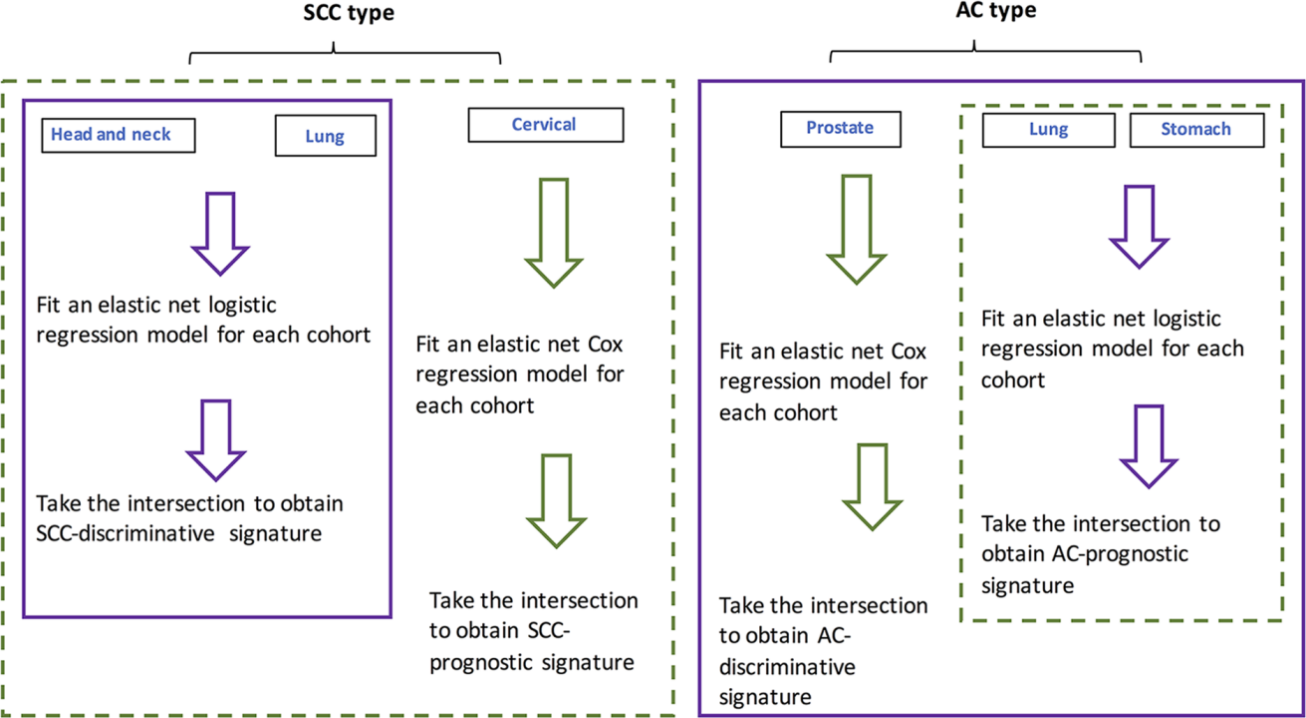
**Flowchart showing the procedure for identifying SCC- and AC-discriminative, and SCC- and AC-prognostic lncRNAs.**

For individual cohorts, elastic-net regression models were fit. The respective intersections of identified lncRNA lists by the models were obtained and deemed as pan-AC discriminative lncRNAs, pan-SCC discriminative lncRNAs, pan-AC prognostic lncRNAs and pan-SCC prognostic lncRNAs. Their performance and biological relevance were investigated subsequently. Figure 4 shows how these four signatures were constructed.

### Pathway enrichment analysis

Connectivity (gene-to-gene interaction) information was retrieved for the target mRNAs by identified lncRNAs from the lncRNADisease 2.0 database [21], and upon those target mRNAs Kyoto Encyclopedia of Genes and Genomes (KEGG) [135] pathway/gene ontology (GO) [136] enrichment analysis was conducted using the String software [137]. A false discovery rate (FDR) of <0.05 was deemed to be statistically significant.

### Performance evaluation

For discriminative values of identified lncRNAs, ridge logistic regression models were fit to estimate the coefficients of identified genes and then probabilities of having tumors were calculated. The receiver characteristic operator (ROC) curves were made, and the area under the ROC curve (AUC) statistics were calculated to evaluate the performance of these signatures.

Multivariate Cox regression models with ridge penalty terms were fit to estimate the coefficients of identified prognostic lncRNAs and calculate the risk scores of death for all patients. The median expression value was used as a cutoff to divide the patients into high-risk group and the low-risk groups. Lastly, log-rank tests were carried out to test if the survival curves of the two groups were the same.

### Statistical language and packages

All statistical analysis was carried out in R version 3.5 (https://www.r-project.org/). Specifically, the Bioconductor org.Hs.eg.db package was used to map ENSEMBL IDs to gene symbols. The glmnet package [138] was used to fit the elastic net and the ridge regression models. The survival and survminer packages were used for making Kaplan-Meier curves and carrying out log-rank tests. ROCR and ggplot2 were used for making ROC curves and calculating AUC statistics.

### Availability of data and materials

LncRNA expression profiles (RNA-Seq data) and the corresponding clinical information were downloaded from the Tanric (The Atlas of ncRNA in Cancer) webpage (https://ibl.mdanderson.org/tanric/_design/basic/download.html) and the Genomic Data Commons (http://www.cbioportal.org/) of TCGA (The Cancer Genome Atlas) database.
